# The Prognostic Factors in Children With Status Epilepticus and Status Epilepticus Severity Score Scales

**DOI:** 10.1155/bn/6660355

**Published:** 2025-01-17

**Authors:** Serap Bilge, Gülen Gül Mert, Özlem Hergüner, Faruk İncecik, Şakir Altunbaşak, Dinçer Yıldızdaş, Özden Özgür Horoz, Duygu Kuşcu

**Affiliations:** ^1^Department of Pediatric Neurology, College of Medicine, Balcalı Hospital, Çukurova University, Adana, Turkey; ^2^Department of Pediatric Intensive Care, College of Medicine, Balcalı Hospital, Çukurova University, Adana, Turkey; ^3^Department of Pediatric Psychology, College of Medicine, Balcalı Hospital, Çukurova University, Adana, Turkey

**Keywords:** mSTESS, prognosis, status epilepticus

## Abstract

**Background:** According to the International League Against Epilepsy (ILAE) 2015 classification, status epilepticus (SE) is a seizure that lasts longer than 5 min or a frequency of more than one seizure within 5 min, without returning to a normal level of consciousness between episodes. In this study, we aimed to evaluate the prognostic factors of SE and compare our patients with those of patients treated internationally with the modified status epilepticus severity score (mSTESS) to determine the reliability of this scoring system.

**Methods:** The medical records of patients aged 1 month–17 years with SE who were treated at Çukurova University–Balcalı Training and Research Hospital between September 2018 and September 2021 and who were followed in the intensive care unit were included in the study.

**Results:** Seventy-two patients were included in this study. The mean age of the patients with SE was 5 years (3–8). The male/female ratio was 34 (47%)/38 (53%). A history of epilepsy was present in 53% of the patients. The most common etiologies behind SE were meningoencephalitis (19%), febrile status (8%), unknown status (12%), and genetic causes (7%). Comorbidities, including developmental delay/intellectual disability, cerebral palsy, hyperactivity, and autism spectrum disorder, were present in 44 (61%) of the patients. The cutoff was ≥ 2 for unfavorable outcomes and 4 for mortality in our patients according to the mSTESS system. The case mortality rate was 1% in our study. Nonconvulsive SE, slowing and abnormal ground on EEG, being stuporous or comatose, having abnormal MRI–CT, and having a comorbid condition were associated with an unfavorable prognosis in SE patients.

**Conclusion:** The mSTESS is a useful and practical scoring system for predicting the prognosis of SE patients. Nonconvulsive SE, slowing and abnormal ground in EEG, being stuporous or comatose, abnormal MRI–CT, and the presence of comorbid conditions indicated poor prognosis of SE in children.

## 1. Background

Status epilepticus (SE) is the most extreme form of seizure that may occur in patients with previous epilepsy or acute disorders of the central nervous system. SE is a medical emergency that may lead to permanent brain damage or death. SE is very rare, and most people with epilepsy will never experience this disorder [1–4]. The estimated annual incidence of SE in children ranges from 10 to 38 per 100,000, and its prevalence is higher in younger children, especially in the first year of life. The estimated mortality in SE patients is 20%. SE can occur as convulsive SE or nonconvulsive SE. Many factors determine the prognosis of this disorder; among these major factors are the duration of SE, patient age, and the underlying cause. Common causes of SE include cerebrovascular disorders, brain trauma, infections, low antiepileptic drug levels in patients with epilepsy, inflammatory causes, inborn errors of metabolism, and genetic causes. In this study, we aimed to determine the prognostic factors for SE in our patients and to test the availability of the modified status epilepticus severity score (mSTESS) system [3–10].

## 2. Methods

The medical records of patients aged 1 month–17 years who were treated at Çukurova University–Balcalı Training and Research Hospital from September 2018 to September 2021 were retrospectively reviewed to detect the following ICD-10 codes: G41.0, grand mal SE; G41.1, petit mal SE; G41.2, complex partial SE; G41.8, other SE; and G41.9, SE unspecified. Patients with the abovementioned ICD codes were included in the study. Demographic and clinical variables, including age, sex, age of onset of SE, type of seizure, etiology, available genetic analysis results, EEG results, magnetic resonance imaging (MRI) results, mSTESS, received treatments, responses to treatments, developmental status at baseline before SE and after SE (before discharge at the last available follow-up visit), and mortality, were recorded [11, 12]. SE was diagnosed according to the International League Against Epilepsy (ILAE) 2015 classification. All noncompliant seizures, psychogenic nonepileptic seizures, and the newborn population were excluded from the study. Patients with severe disabilities and children with acute hypoxic–ischemic encephalopathy were also excluded from the study. Approval from the institutional ethics committee was obtained. Written informed consent was obtained from the parents/caregivers.

### 2.1. Procedure

The children who were enrolled in our study were treated according to the standard international protocol (the typical protocol for the management of SE in our institute). The antiseizure medications (ASMs) were considered to be effective if there was a cessation of SE within 10 min of the initial dose of medication and if there was a sustained absence of convulsions for 30 min. The response of the patients to the ASMs was also noted. A patient was classified as having benzodiazepine (BZD)-responsive SE if the SE responded to a first or second dose of BZD. SE was defined as SE that responded to second-line ASM after BZD, which usually involved phenytoin/valproic acid/levetiracetam. Refractory SE was defined as SE persisting despite the administration of two appropriate anticonvulsants (BZD and phenytoin) at acceptable doses and responding only to third-line ASM involving a midazolam infusion or anesthetic agent infusion. Super-refractory SE was defined as SE that continued for 24 h or more after the onset of anesthesia infusion, including those cases in which the SE recurred upon the reduction or withdrawal of anesthesia [11–15]. All of the patients with SE were followed until discharge or death (which represents the protocol of SE in our hospital). At least 1 h of EEG was performed for all of the patients with SE, refractory SE, or super-refractory SE, as well as for patients with suspected nonconvulsive SE. EEGs were recorded by using 20 channels, including 19 standard scalp electrodes. The EEG signal was sampled at 256 Hz with a low-frequency filter at 1 Hz and a high-frequency filter at 70 Hz. The scalp EEG signal was then displayed in a bipolar anteroposterior montage. EEG was repeated as per the clinical indications. The EEG machine was a Nihon Koden Neurofax EEG 1200.

### 2.2. Study Design

The suspected causes of epilepsy in patients, such as cerebrovascular disorders, brain trauma, infections, low antiepileptic drug levels, inflammatory causes, inborn errors of metabolism, and genetic causes, were recorded. If there was no obvious cause, the etiology was recorded as unknown.

The seizure type was categorized as focal, generalized, focal with secondary generalization, or nonconvulsive SE considering the classification proposed by the ILAE. EEG results were retrieved from reports by the attending epileptologist, and the EEGs that were performed closest to the date of the SE episode were included in the study. EEG abnormality types were classified into the following groups:

1. Normal
2. Spikes, spike waves, and other epileptiform abnormalities
3. Slowing, abnormal ground, and other nonepileptiform activities

Similarly, MRI results were acquired by using the radiologist's reports in the medical records. Results of MRI (1.5 T GE MR machine) that was performed closest to the date of the SE episode were included in the study.

A child was considered to be developmentally delayed at baseline if there was explicit information about delayed milestones that were mentioned by the physician's assessment. Patients were followed until the last available follow-up date by a pediatric neurologist. Neurocognitive outcomes were evaluated based on psychotechnics (Denver-II/Stanford–Binet test or Wechsler Intelligence Scale for Children–Revised (WISC-R) measurements performed by a pediatric psychologist; however, some children were assessed by a pediatric neurologist, and detailed examinations regarding muscle strength, speech abnormality–related cognitive behaviors, and awareness of places around various places were recorded in the medical files. In children with premorbid developmental delay or disability, a return to their baseline functional status was considered a favorable outcome, and any decline was considered unfavorable. Moreover, disabilities were assessed (if there was no medical record of the patient before SE in our health center) by asking the parents about the baseline functional status of their children and asking them to show videos of their children before SE, in which the ability of these children to walk, run, speak, and answer questions (if available) could be observed.

The most commonly used scoring system for determining the prognosis of convulsive and nonconvulsive SE patients is the mSTESS or the status epilepticus pediatric severity score (STEPSS) (assessment score ranging from 0 to 6) Table 1. The STEPSS parameters were noted at the time of presentation in the emergency room or the intensive care unit (ICU). The level of consciousness was noted before BZD administration [11].

### 2.3. Statistical Analysis

Categorical variables are expressed as numbers and percentages, whereas continuous variables are summarized as the mean and standard deviation and as the median and interquartile range (IQR), where appropriate. To compare categorical variables between the groups, Pearson's chi-square test or Fisher's exact test was used, depending on whether the expected value problem was evident. The normality of the distribution of continuous variables was confirmed with the Shapiro–Wilk test. For the comparison of continuous variables between the groups, Student's *t*-test or the Mann–Whitney *U* test was used, depending on whether the statistical hypotheses were supported. Multiple logistic regression analysis was performed to determine significant predictors of outcome. Variables significant at the *p* < 0.25 level in the univariate analyses were used in the logistic regression analysis. Receiver operating characteristic (ROC) curve analysis was performed to identify the optimal cutoff point for the mSTESS to predict the outcome. All of the analyses were performed by using the IBM SPSS Statistics version 20.0 statistical software package. The statistical level of significance for all of the tests was considered to be 0.05.

## 3. Results

**Figure 1:**
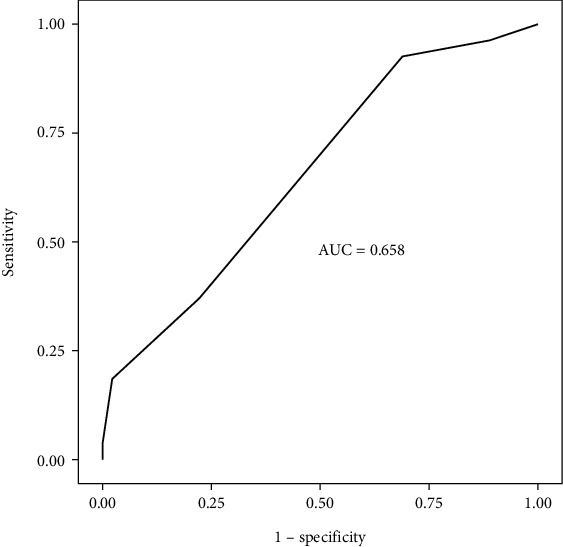
ROC curves depicting the prediction accuracy of mSTESS (in all patients) for prognosis.

Seventy-two patients were included in this study. The mean age of the patients with SE was 5 years [3–8]. The male-to-female ratio was 34 (47%)/38 (53%). A history of epilepsy was present in 53% of the patients (Table 2). The most common etiologies underlying SE were meningoencephalitis (19%), febrile status (8%), unknown (12%), and genetic causes (7%). Tables 2, 3, and 4 show the characteristics of the patients, the etiology underlying SE (Table 2), and factors that played a role in the good and poor prognoses of SE (Table 4). Comorbidities, including developmental delay/intellectual disability, cerebral palsy, hyperactivity, and autism spectrum disorder, were present in 44 (61%) of the patients with SE. The case mortality rate was 1% in our study. ROC curves depicting the prognostic accuracy of mSTESS (in all of the patients) were generated (Figure 1). The classification accuracies of mSTESS according to different cutoff points and the predictive accuracy of the mSTESS score are also shown (Tables 5 and 6).

## 4. Discussion

SE is a common neurological emergency. The incidence of short-term mortality ranges from 0.9% to 3.6% in children. The most vulnerable structure to seizures is the hippocampus, which is involved in learning and memory. Other structures that demonstrate necrosis following SE include the amygdala, dorsomedial thalamic nucleus, medial layers of the neocortex, and cerebellum; thus, SE has a destructive effect on cognitive, motor, and sensory functions [1–4]. SE outcome is mainly determined by any delay in treatment, the refractory degree of the seizures, the underlying etiology, and the response to treatment. The determination of the factors that can predict the outcome of patients with SE is highly important because these factors may be useful and essential for deciding on any further treatment that can directly affect patient prognosis [12–15].

Age is a strong indicator of outcome in SE patients. Febrile seizures and acute symptomatic etiologies are most common in children younger than 2 years, whereas cryptogenic and remote symptomatic etiologies are more common in older children. According to some studies, mortality due to SE is high in children younger than 1 year, which is possibly due to the fact that the brain is in a period of rapid development at that age [16]. Our study demonstrated that age did not play a role in the prognosis of SE, which was likely due to the fact that most of our patients younger than 2 years had SE due to acute symptomatic etiologies such as meningoencephalitis, febrile SE, and an unknown origin. The effect of age could be due to the fact that the etiology of SE differs with age. Sex can also affect the prognosis of patients with SE. According to a study conducted on adult patients to assess the role of gender in SE, the hospital-based prevalence of SE appeared to be greater for men than women. The hospital stays of men with SE were shorter than those of women, which indicates that the severity of SE tends to be greater in females who require prolonged hospitalization [17]. In our study, the mean age group was 5 years, and sex did not play a role in the prognosis of SE patients. This may be because the estrogen and testosterone hormones do not affect young males or females differently as it does in males or females after puberty.

Ethnicity is also a factor that can play a major role in prognosis of this disorder. There is a higher incidence of SE and lower mortality among black individuals. This could be related to the underlying illness, access to medical care, compliance, or other intrinsic biological factors. In our study, all of the included patients were Asian; therefore, such a comparison could not be performed [18].

An impaired level of consciousness at SE onset is independently associated with mortality. In a recent retrospective population-based study of adult patients with SE in Salzburg, impaired consciousness was associated with a high case fatality rate of 33%, compared with 8.2% in awake patients [18, 19]. In our study, 47% of the patients (alert, confused, and somnolent) were in the good prognosis group, and 4% were in the poor prognosis group, whereas 53% of the patients (comatose and stuporous) were in the good prognosis group, and 96% were in the poor prognosis group. This scenario could be explained by the destruction of brain cells due to prolonged seizures. Semiology could also play a role in the prognosis of SE patients. According to a previous study, generalized myoclonic SE is associated with poor outcomes and high mortality. We have noted from the STESS that generalized seizures have a worse prognosis than focal seizures. Our study matches these results [18, 19].

It is difficult to determine the exact duration of SE because its onset is frequently not known. Overall, a longer duration of SE was associated with greater mortality. However, new-onset refractory SE can be associated with significant recovery even after a prolonged duration of SE. In our study, the duration of SE could not be obtained from the medical files; however, we followed a male patient aged 10 years with SE in the ICU due to febrile infection-related epilepsy syndrome (FIRES). He had very prolonged durations and frequent SE despite the fourth-line treatment protocol for SE. Furthermore, he had no seizure-free periods, and he died 9 months after admission to the ICU due to complications.

Overall, EEG findings were less important than the duration of SE and etiology as predictors of outcomes. The EEG pattern is nonspecific in children with SE. In a systematic review of adult patients with SE, periodic epileptiform discharges (PEDs) were found to be associated with poor outcomes. According to an EEG study of 50 patients, PEDs were associated with greater mortality/vegetative state (44%) than were non-PEDs (19%). PEDs mainly occur due to diffusion restriction in the cortical area, which is commonly observed in SE. Normalization of the EEG after SE was correlated with good outcomes [18, 19]. In our study, patients with slow-wave EEGs had a worse prognosis than patients with normal EEGs or with spikes and/or sharp waves.

Huang et al. studied 15 patients with SE via MRI. These MRI abnormalities consisted of decreased diffusion in diffusion-weighted imaging (DWI**)**/apparent diffusion coefficient (ADC), and increased signal in T2 MRI sequences was usually accompanied by focal cerebral edema and increased vascularization [20–25]. In our study, seven (9.7%) patients had increased T2 hyperintensity and decreased diffusion in the ADC. The failure of the Na/K ATPase pump may lead to cellular sodium and water influx, thus resulting in cytotoxic edema. Other mechanisms may include the excessive release of excitatory amino acids such as glutamate and increased membrane ion permeability [16–19]. In our study, it was concluded that the presence of abnormal MRI–CT results was associated with poor outcomes in children with SE.

Metabolic imbalance plays a crucial role in prognosis. A decrease in glucose and glycogen with parallel increases in lactate indicates a high rate of glycolysis, and seizures can result in profound elevations in serum lactate. In a study conducted by Sop et al. on adult patients with seizures regarding the relationship between high blood lactate levels, mortality, and poor prognosis, no relationship could be established. In our study, we concluded that patients with a poor prognosis mainly had a higher lactate level, yet the mean average blood lactate level was less than 2 mmol/l, which can be considered normal because in other studies, when high and low lactate levels were classified, a cutoff of 2 mmol/L was used as an indicator of a high lactate level. The difference between the results of these studies could be due to many factors, such as the time at which blood samples were obtained, as well as the fact that our study population involved a pediatric group and the presence of other comorbidities, such as infections, which could have affected blood lactate levels [26, 27].

Associated conditions can always complicate the course of SE and are considered important prognostic factors for cardiac arrhythmia, cardiac damage because of catecholamine surge, and respiratory failure. The presence of these complications can also affect the prognosis of patients with SE. In our study, there was no effect of low blood pressure, arrhythmia, or intubation on poor prognosis; however, in our study, there was a male patient aged 10 years followed by FIRES who experienced low blood pressure, arrhythmia, intubation, and multiple organ failure. He died 9 months after admission to the pediatric ICU. Moreover, he experienced super-refractory SE despite the fourth-line treatment protocol for SE, which included anakinra ketogenic diet and cannabidiol treatment [28, 29].

Comorbidities such as cerebrovascular disease, renal failure, hyperactivity, and autism can coexist with SE in children. These comorbidities can affect the prognosis of patients with SE. Most of the studies researching the effect of comorbidities on the prognosis of SE have been conducted in adult patients. Alvarez et al. reported that comorbidities likely affect the outcome of SE in a relatively marginal way. The comorbidities of the adult patients in that study mainly included cerebrovascular disease. The presence of comorbidities does not necessarily predict a poor outcome [28, 29]. In our study, comorbid conditions (autism/hyperactivity/liver disease/renal disease/cerebrovascular disease) were detected in 46 (61%) of the total SE patients, and 26 (96%) of the SE patients had a poor prognosis. Comorbidities are important because of the contraindications and side effects of antiseizure drugs. In this regard, they may influence the outcome by influencing the treatment protocol.

STESS has been proven to be a good predictor of morbidity and mortality, and there is an urgent need for aggressive treatment. mSTESS was tested in a study in India by Sidharth et al. One hundred forty children (mean age: 5.8 years) were enrolled in this study. Seven children died, and 15 children overall had unfavorable outcomes. The predictive accuracy of the mSTESS was at a cutoff of ≥ 3 for unfavorable outcomes [11]. In our study, this cutoff was ≥ 2 for unfavorable outcomes and ≥ 4 for mortality. The difference of 1 point between the two studies could be due to the different groups of patients in the two studies. In our study, the mean age of the patients was 5.3 years, the percentage of male patients to female patients was almost the same, and the percentage of patients with previous epilepsy was 53%, which is similar to the variability observed in the study by Sidharth et al., except for the male/female ratio, which was greater in the study by Sidharth et al. Furthermore, the three most common SEs in our study were meningoencephalitis (19%), febrile status (8%), unknown (12%), and genetic causes (7%). In the study by Sidharth et al., the most common etiology was acute symptoms, which were present in 25.7% of patients, followed by remote symptoms, which were present in 25%, and febrile SE, which was present in 18.6%. The percentage of different etiologies between the two studies may play a role in producing different values for unfavorable outcomes.

## 5. Conclusion

The mSTESS is a useful and practical scoring system for predicting the prognosis of SE patients. Nonconvulsive SE, slowing and abnormal ground in EEG, being stuporous/comatose, abnormal MRI–CT, and the presence of comorbid conditions favored poor prognosis in SE patients in children.

### 5.1. Limitations of the Study

The performance of a retrospective study is considered a limitation of the study.

## Figures and Tables

**Table 1:** mSTESS/STEPSS system [11].

Alert or somnolent/confused	0
Stuporous or comatose	1
Simple partial, complex partial, absence, myoclonic	0
Generalized convulsive	1
Nonconvulsive, status epilepticus in coma	2
≥ 2 years	0
< 2 years	2
History of previous seizures	
Yes	0
No	1

**Table 2:** Characteristics and variabilities of the patients with SE.

**Parameters**	**Characteristics**
Age (year), median (IQR)	5 (3–8)
Gender, male/female, *n*	3 (47%)/38 (53%)
Previous epilepsy, *n* (%)	38 (53%)
SE semiology, *n* (%)	
Focal	10 (14%)
Generalized	50 (69%)
Nonconvulsive	12 (17%)
The most common etiology, *n* (%)	
Meningoencephalitis	14 (19%)
Genetic	5 (7%)
Unknown	9 (12%)
Lactate, median (IQR)	1.2 (0.9–1.8)
Intubation, *n* (%)	7 (10%)
EEG, *n* (%)	
Normal	15 (21%)
Epileptic	42 58(%)
Slowing/abnormal ground	15 (21%)
Convulsive/nonconvulsive, *n*	59/12
Consciousness *n* (%)	
Alert/somnolent/confused	22 (31%)
Stupor/comatose	50 (69%)
Prognosis, *n* (%)	
Good prognosis	45 (62%)
Poor prognosis	27 (38%)
mSTESS, median (IQR)	2 (2–3)
Third-line treatment of SE protocol (infusion of anesthetic agents), *n* (%)	43 (60%)
Low blood pressure, *n* (%)	15 (21%)
Arrhythmia, *n* (%)	1 (1%)
MR tomography abnormal findings, *n* (%)	44 (61%)
T2 hyperintensity increase and decreased ADC	7 (9.7%)
Baseline development (before SE), *n* (%)	41 (58%)
Development at follow-up (after SE), *n* (%)	
Regression	26 (36%)
Stable	46 (64%)
Comorbidity, *n* (%) autism/hyperactivity/liver disease/renal disease/cerebrovascular disease	44 (61%)
Mortality, *n* (%)	1 (1%)
The patients were assessed by a pediatric psychologist using a psychotechnique measure	60 (83%)

**Table 3:** The etiology of patients with SE.

Febrile status	6 (8%)
Meningoencephalitis	14 (19%)
Genetic	5 (7%)
Unknown	9 (12%)
Accidents/intoxications	6 (8%)
Epilepsy and irregular use of antiseizure medication or sudden stoppage	5 (7%)
Metabolic	5 (7%)
PRES	3 (4%)
Intracranial bleeding/trauma	4 (6%)
Dravet syndrome/epileptic encephalopathy	5 (7%)
LGS and West syndrome due to CMV	2 (3%)
Structural anomaly	5 (7%)
FIRES	1 (1%)
Stroke—Schimke immuno-osseous dysplasia	1 (1%)
TSC	1 (1%)

Abbreviations: FIRES: febrile infection-related epilepsy syndrome; LGS: Lennox–Gastaut syndrome; PRES: posterior reversible encephalopathy syndrome; TSC: tuberous sclerosis complex.

**Table 4:** Factors that influence the prognosis of patients with SE.

	**Good prognosis**	**Poor prognosis**	**p**
Age (year), median (IQR)	4 (2–7)	6 (3–10)	0.112
Gender *n* (%)			
Male	21 (47%)	13 (48%)	0.903
Female	24 (53%)	14 (52%)	
Previous epilepsy, *n* (%)	23 51(%)	15 (56%)	0.715
SE semiology, *n* (%)			
Focal	8 (18%)	2 (7%)	
Generalized	34 (76%)	16 (59%)	**0.014**
Nonconvulsive	3 (7%)	9 (33%)	
The most common etiology, *n* (%)			
Meningoencephalitis	10 (22%)	2 (7%)	
Febrile status	6 (13%)	1 (4%)	0.383
Genetic	5 (11%)	0 (0%)	
Unknown	6 (13%)	3 (11%)	
Lactate, median (IQR)	1.1 (0.8–1.2)	1.8 (1.6–1.9)	**< 0.001**
EEG, *n* (%)			
Normal	13 (29%)	2 (8%)	
Epileptic	26 (58%)	16 59(%)	**0.030**
Slowing/abnormal ground	6 (13%)	9 (33%)	
Convulsive/nonconvulsive, *n* (%)			
Convulsive	41 (93%)	18 67(%)	**0.007**
Nonconvulsive	3 (7%)	9 (33%)	
Consciousness, *n* (%)			
Alert, confused, somnolent	21 (47%)	1 4(%)	**< 0.001**
Stupor, comatose	24 (53%)	26 (96%)	
mSTESS, median (IQR)	2 (1–2)	2 (2–3)	**0.016**
Third-line treatment of antiseizure (infusion of anesthetic agents), *n* (%)	25 (56%)	18 (67%)	0.352
Low blood pressure, *n* (%)	10 (22%)	5 (19%)	0.708
Intubation, *n* (%)	2 (4%)	5 (19%)	0.095
Arrhythmia, *n* (%)	0 (0%)	1 (4%)	0.375
MRI–CT abnormal findings, *n* (%)	22 (49%)	22 (81%)	0.006
Baseline development (before SE), *n* (%)	29 (66%)	12 (44%)	0.075
Morbidity, *n* (%)	18 (40%)	26 (96%)	**< 0.001**

*Note:* The bold values indicate that these factors play a role in poor prognosis; values less than 0.05 are statistically meaningful and important. According to the comparisons made, it was found that patients with poor prognosis had higher lactate levels, higher rates of patients with nonconvulsive status epilepticus and stupor, and lower rates of patients with normal EEG findings but higher rates of patients with disturbed background and associated morbid conditions.

**Table 5:** The classification accuracies of mSTESS according to different cutoff points.

**mSTESS**	**Sensitivity**	**Specificity**	**C**
≥ 0	1.00	0.00	0.38
≥ 1	0.96	0.11	0.43
≥ 2	0.93	0.31	0.54
≥ 3	0.37	0.78	0.63
≥ 4	0.19	0.98	0.68
≥ 5	0.04	1.00	0.64
	0.00	1.00	0.63

**Table 6:** Predictive accuracy of mSTESS.

**Score**	**The area under the ROC curve**	**Cutoff**	**Sensitivity**	**Specificity**	**PPV**	**NPV**
mSTESS	0.658 (95% CI: 0.53–0.79)	**≥ 2**	0.93	0.31	0.45	0.88

*Note:* The bold value indicates the poor prognosis of the m-STESS score.

## Data Availability

Data are available at Ass Prof. G.G.M.'s repository. The datasets used and/or analyzed during the current study are available from the corresponding author upon reasonable request.

## Nomenclature

SE: status epilepticus
ILAE: International League Against Epilepsy
mSTESS: modified status epilepticus severity score
BDZR: benzodiazepine drug resistant
RSE: resistant status epilepticus
DWI: diffusion-weighted MR
ADC: apparent diffusion coefficient
PEDs: periodic epileptiform discharges
NCSE: nonconvulsive status epilepticus
